# Reeve’s Muntjac (*Muntiacus reevesi*) Habitat Suitability Under Climate Change Scenarios in Hupingshan National Nature Reserve, China

**DOI:** 10.3390/ani15020160

**Published:** 2025-01-09

**Authors:** Qi Liu, Jianyang Ye, Zujie Kang, Guiqing Yu, Cuncun Yang, Jianjun Li, Tao Tang

**Affiliations:** 1 College of Computer and Mathematics Computer, Central South University of Forestry and Technology, Changsha 410004, China; lq20221200534@163.com (Q.L.); lijianjun_21@163.com (J.L.); 2 College of Forestry, Central South University of Forestry and Technology, Changsha 410004, China; yejianyang1@126.com; 3 Key Laboratory of National Forestry and Grassland Administration on Forest Resources Management and Monitoring in Southern Area, Changsha 410004, China; 4 Hupingshan National Nature Reserve Management Bureau, Changde 415319, China; hpskzj@126.com (Z.K.); yz7009@126.com (G.Y.); y648320387@163.com (C.Y.)

**Keywords:** *Muntiacus reevesi*, suitability habitat, south china tiger, MaxEnt, climate change

## Abstract

Climate change and human disturbance are critical factors affecting the habitat distribution of wild animals, with implications for management strategies such as protecting migration corridors, habitat restoration, and species conservation. In the Hupingshan National Nature Reserve (NNR), Reeve’s muntjac (*Muntiacus reevesi*) is a key prey species for the South China tiger (*Panthera tigris amoyensis*), which is extinct in the wild and targeted for reintroduction by the Chinese government. Thus, understanding the habitat distribution and abundance of Reeve’s muntjac is essential to ensure the survival and sustainability of reintroduced tiger populations. Despite significant conservation efforts, the impacts of climate change and human disturbance on Reeve’s muntjac habitat distribution in Hupingshan NNR remain unclear, though these factors could necessitate adaptive tiger management strategies due to shifts in prey abundance. In this study, we employed an optimized MaxEnt model to assess current habitat distribution and identify key environmental variables influencing the habitat distribution of Reeve’s muntjac. Assuming non-climatic environmental factors will remain constant over the next century, we projected future habitat distribution under two shared socioeconomic pathways (SSP126 and SSP585) for the mid-century (2050s) and the late-century (2090s). Comparative analyses of current and projected habitat areas revealed potential impacts of climate change on this species. MaxEnt outputs classified habitat suitability into high, medium, and low levels. Results showed that climatic and human disturbance factors contributed 35.2% and 49.4% to habitat suitability, respectively. Under the SSP126 scenario, habitats with decreased suitability covered 0 km^2^ in the 2050s, expanding slightly to 4.2 km^2^ in the 2090s, while those with increased suitability spanned 491.1 km^2^ (2050s) and 463.2 km^2^ (2090s). Under the SSP585 scenario, habitats with decreased suitability covered 10.2 km^2^ in the 2050s and 431.8 km^2^ in the 2090s. Habitats with increased suitability were comparatively smaller under SSP585, covering 162.0 km^2^ (2050s) and 1.1 km^2^ (2090s). These findings suggest that while mid-century climate projections (SSP126 and SSP585) may support Reeve’s muntjac’s survival, habitat loss is projected by 2090s (SSP126). Thus, future climate change may lead to decreased habitat suitability and increased fragmentation, raising extinction risks for Reeves’s muntjac. Mitigating these effects could involve establishing migration corridors, minimizing human disturbances, and potentially supplementing prey populations with captive-bred prey. Such measures are essential to support the South China tiger reintroduction plan and help ensure that prey availability remains sufficient for sustaining reintroduced tiger populations.

## Simple Summary

Reeve’s muntjac (*Muntiacus reevesi*), a key prey species of the South China tiger (*Panthera tigris amoyensis*) in the Hupingshan National Nature Reserve, plays a crucial role in supporting tiger reintroduction efforts. We applied the MaxEnt model to assess Reeve’s muntjac’s current and future habitat distribution under two climate scenarios (SSP126 and SSP585) for the mid-century (2050s) and the late-century (2090s), respectively. The results showed that climate (35.2%) and human activities (49.4%) played a key role in the habitat distribution of Reeve’s muntjac. Currently, the high, medium, and low suitability habitats were 150.9 km^2^, 290.9 km^2^, and 225.9 km^2^, respectively. The high and medium suitability habitat area will ascend to 658 km^2^ (2050s) but slightly descend to 651.3 km^2^ (2090s) under SSP126. The high and medium suitability habitat area will ascend to 540.7 km^2^ (2050s) but descend to 73.6 km^2^ (2090s) under SSP585. These findings suggest that while mid-century conditions may support the species, late-century climate conditions, particularly under SSP585, could cause severe habitat loss for Reeve’s muntjac. Conservation measures such as establishing migration corridors and minimizing human activities are essential to mitigate these impacts and support both the survival of Reeve’s muntjac and the success of the South China tiger reintroduction plan.

## 1. Introduction

Global environmental challenges, particularly climate change and human activities, profoundly affect the geographical distribution, population size, and feeding behaviors of wild animals, accelerating species extinction rates [1]. In response, many species have already shifted their range boundaries to track habitats with suitable conditions [2]. However, the rapid pace of climatic changes and human activities is making it increasingly difficult for species to adapt to their preferred habitats [3].

The Reeve’s muntjac (*Muntiacus reevesi*) is a small forest-dwelling deer widely distributed across Southern China, particularly favoring mountainous terrain with dense cover [4]. Although classified as of “least concern” by the International Union for Conservation of Nature (IUCN Red List 2024, https://www.iucnredlist.org/, accessed on 9 June 2023), the population of Reeve’s muntjac is experiencing a decline due to threats, such as habitat loss from human activities, i.e., agricultural expansion, commercial and residential infrastructure development, logging, and poaching [5]. These factors underscore the urgency of understanding its habitat requirements to inform effective conservation strategies.

Habitat availability is critical for species survival and reproduction, and understanding habitat distribution patterns and utilization characteristics is fundamental for formulating species protection and management strategies [6]. Protecting habitats serves as an effective measure for conserving wildlife populations [7]. While systematic field surveys can provide valuable information on species’ habitat preferences [8], they are often time- and resource-intensive and challenging to conduct [9], especially for elusive species such as Reeve’s muntjac, which exhibit shy, solitary behavior, and agility and inhabit remote, rugged areas [10]. To address these challenges, species distribution models (SDMs) have been widely applied to identify suitable habitats for target species within complex landscapes [11,12,13,14]. Among these, the maximum entropy (MaxEnt) model integrates species occurrence points of wild species with environmental variables, such as climate and topography, to estimate current and future habitat distributions over time [15,16]. MaxEnt requires relatively simple data, operates efficiently, and performs well with limited sample sizes [15]. In addition, this model enables projections of computational results across temporal or spatial domains, allowing for predictions of both actual and potential habitat distributions [17]. This model has been extensively applied in diverse contexts, including forecasting future ranges of invasive species [18], identifying priority areas for nature reserves [19], and assessing the impacts of climate change on biodiversity [20].

The MaxEnt model has also been applied to estimate suitable habitats for Reeve’s muntjac [4,10,16]. For instance, Sun et al. [4] employed the MaxEnt model, incorporating species distribution records from previous studies along with elevation and nine climatic variables, including mean diurnal air temperature range (BIO2), isothermality (BIO3), and mean temperature of the driest quarter (BIO9), to assess and predict the habitat distribution of Reeve’s muntjac in southern China. Their findings indicated that the historical distribution of Reeve’s muntjac was highly sensitive to climate change, and future projections suggested a net increase in suitable habitat areas under changing climatic conditions. Similarly, Meng et al. [12] used MaxEnt, alongside infrared camera data, climate variables including BIO2 and BIO3, and four additional environmental factors, to model the current habitat distribution of Reeve’s muntjac in Fanjingshan National Nature Reserve (NNR), revealing their preference for areas with a BIO2 range of 7.0–7.3 °C and a BIO3 range of 25.2–26.0 °C. In another study, Freeman et al. [16] employed R 4.4.1 software to simulate suitable habitats for Reeve’s muntjac across Great Britain and Ireland by incorporating species records, bioclimatic variables such as annual mean temperature (BIO1), annual precipitation (BIO12), and topographical parameters such as altitude and slope. Their analysis identified BIO1 and BIO12 as the most influential factors, with an overall median permutation importance of approximately 70%. Collectively, these studies underscore the critical role of climate data in forecasting habitat shifts and inform conservation strategies aimed at mitigating extinction rates.

The Hupingshan NNR, a key conservation area in central-south China, is home to a high density of Reeve’s muntjac. In addition, the NNR is an existing protected area of sufficient size within the historical range of South China tigers (*Panthera tigris amoyensis*) to support a small wild population [21,22]. The South China tiger, a tiger subspecies native to China [23], is considered likely extinct in the wild [24,25]. To restore a viable wild population, the Chinese government has developed a strategic plan to reintroduce South China tigers into Hupingshan NNR, using founders from captive-bred populations [25,26]. Studying tiger populations in forested environments is particularly challenging due to their low densities, extensive range requirements, and elusive behavior—challenges that are further complicated when the species is extinct in the wild. Consequently, researchers can examine prey populations, as predator-prey interactions are well-documented, with activity patterns of tigers closely aligning with those of their prey species [24,26,27]. Within this ecosystem, the Reeve’s muntjac is recognized as a crucial prey for the South China tiger [28]. Therefore, assessing the habitat distribution and availability of Reeve’s muntjac is essential for supporting the survival and sustainability of the reintroduced tiger population.

In this study, we developed a MaxEnt model to assess the current and future habitat distribution of Reeves’s muntjac in Hupingshan NNR, with a focus on identifying the environmental variables that influence habitat selection. Moreover, we examined the potential impacts of climate change on habitat availability, comparing habitat distribution patterns across different time periods and climate scenarios. We further addressed the following research questions: (1) What is the current habitat distribution pattern of Reeves’s muntjac in Hupingshan NNR? (2) Which and how environmental factors significantly influence their habitat selection? (3) How is climate change expected to influence future habitat availability? By addressing these questions, we aimed to provide critical insights for the conservation of Reeves’s muntjac and inform strategies for the successful reintroduction of the South China tigers in Hupingshan NNR.

## 2. Materials and Methods

### 2.1. Study Area

**Figure 1:**
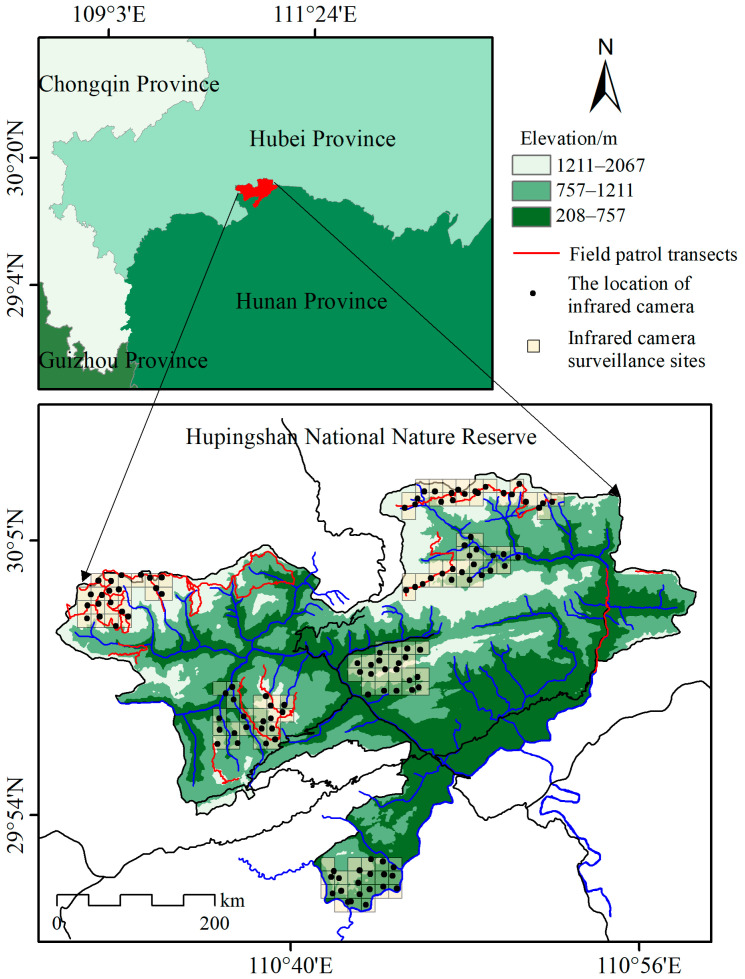
Map showing the locations of infrared camera surveillance sites, infrared camera placements, and field patrol transects for Reeves’s muntjac in Hupingshan National Nature Reserve.

Hupingshan NNR is located in the northernmost part of Shimen County, Hunan Province, bordering Hefeng County and Wufeng Tujia Autonomous County in Hubei Province (Figure 1; area: 667.7 km^2^; 110.4833° to 110.9833° E; 29.8333° to 30.1500° N) [29]. Recognized as one of the 200 globally significant ecological regions by the World Wide Fund for Nature (WWF), the Hupingshan NNR and its surrounding area have critical conservation value [28]. The reserve lies in the upper reaches of the Xie River—the second-largest tributary of the Lishui River in the Dongting Lake region [30]. The terrain of the reserve is dominated by east–west-oriented mountain ranges with steep topographical variation, with altitudes ranging from 208 m to 2075 m [31]. The forest cover in the reserve is remarkably high, with a coverage rate of 90.1%, and exhibits distinct vertical stratification of vegetation types based on altitude [32]. At elevations below 1100 m, the area is dominated by subtropical evergreen broad-leaved forests. Between 1100 m and 1500 m, mixed evergreen and broad-leaved forests are common. From 1500 m to 1700 m, deciduous broad-leaved forests prevail, while shrubs and alpine meadows dominate areas above 1750 m [21]. The reserve experiences a subtropical montane climate, with an annual mean temperature of 9.2 °C and a mean annual precipitation of 1899 mm [33]. Soils in the reserve exhibit pronounced vertical differentiation. At elevations below 600 m, the soils are predominantly red or purple in color. Between 600 m and 1400 m, yellow-brown or yellow soils are prevalent, while above 1400 m, the soils are typically gray-brown mountainous soils [34]. The unique combination of high biodiversity, distinct vegetation zones, and varied topography makes Hupingshan NNR a critical habitat for a variety of ungulate species such as Reeve’s muntjac, wild boar (*Sus scrofa*), tufted deer (*Elaphodus cephalophus*), and Chinese goral (*Naemorhedus griseus*) and an ideal site for reintroduction efforts of the South China tiger.

### 2.2. Data and Sources

Between October 2020 and October 2021, 120 infrared cameras and 31 patrol transects were deployed across Hupingshan NNR to monitor Reeves’s muntjac activity (Figure 1). We established six infrared camera sites. Each site consisted of 20 contiguous 1 km × 1 km grids (20 km^2^ per site), totaling a monitored area of 120 km^2^. Additionally, we set eight monitoring stations based on the vegetation and topography of each station and 25 patrol line transects, each following the mountain ridges. Each transect was 5–9 km in length, averaging 7 km, with a single-side width of 25 m. After filtering out consecutive photos of the same individuals at the same locations, a total of 2340 valid photos were obtained, accurately identifying the species. These occurrence points were used as species distribution data [13].

To model the habitat distribution of Reeve’s muntjac, we selected environmental variables based on previous research [4,11,13,14,16,35,36]. These variables can be divided into five categories: vegetation, topographic, human disturbance, hydrological, and climatic factors. Vegetation factors were represented by the normalized difference vegetation index (NDVI), obtained from the Data Center for Resources and Environmental Sciences at the Chinese Academy of Sciences (http://www.resdc.cn/, accessed on 9 June 2023). Topographic factors included elevation, slope, and aspect, derived from the digital elevation model (DEM; GDEMV2 30 m), and downloaded from the Geospatial Data Cloud (http://www.gscloud.cn/, accessed on 9 June 2023). Human disturbance factors comprised distance from roads, distance from settlements, land use and cover change (LUCC), gross domestic product (GDP) at 1 km resolution, and population density (POP) at 1 km resolution. Data on roads, including primary, secondary, tertiary, and residential roads, were obtained as vector files from OpenStreetMap (https://www.openstreetmap.org/, accessed on 9 June 2023). The distances to these roads—distance to primary roads (DTP), distance to secondary roads (DTS), distance to tertiary roads (DTT), distance to residential roads (DTR), and distance to government residences (DTG)—were computed using the Euclidean distance function in ArcGIS 10.4 (ESRI, Inc., Redlands, CA, USA), with a spatial resolution of 30 m × 30 m. In addition, LUCC data for the year 2020 (30 m resolution), alongside GDP and POP (both with a resolution of 1 km), were sourced from the Data Center for Resources and Environmental Sciences at the Chinese Academy of Sciences (http://www.resdc.cn/, accessed on 9 June 2023). Hydrological factors included distance to rivers (DTRI) and distance to water bodies (DTWB). Vector data for both water bodies and rivers were sourced from OpenStreetMap (https://www.openstreetmap.org/, accessed on 9 June 2023) and supplemented with information sourced from the Hupingshan NNR Management Bureau and the LUCC dataset, respectively. The Euclidean DTRI and DTWB were calculated using the Euclidean distance function in ArcGIS, with a spatial resolution of 30 m × 30 m. Climatic factors included 19 variables, such as annual mean temperature, mean annual precipitation, and precipitation of the coldest quarter [37].

We selected the BCC-CSM2-MR model from the CMIP6 suite of the World Climate Center, which is recognized as the most suitable climate change simulation for China [38]. Climatic factors for the current period (1970–2000a) and two future periods (2050s: 2040–2060a, 2090s: 2080–2100a) were obtained from the WorldClim database (https://www.worldclim.org/, accessed on 9 June 2023) at a spatial resolution of 1 km. These CMlP6-downscaled future climate projections incorporate four shared socioeconomic pathways (SSPs): 126, 245, 370, and 585 [39]. In this study, we predicted the habitat distribution of Reeves’s muntjac for the 2050s and 2090s under two future climate scenarios: SSP126 and SSP585. A total of 33 environmental variables influencing the geographic distribution of Reeves’s muntjac were considered (Table 1), ensuring that the key factors affecting its spatial distribution were thoroughly considered.

All datasets were integrated and processed using ArcGIS 10.8 (ESRI, Inc.). The thematic layers were projected onto the Universal Transverse Mercator (UTM) zone 49N with the WGS1984 datum. Subsequently, these layers were converted to raster grids with a cell size of 30 m, matching the original resolution of the LUCC dataset.

### 2.3. Data Preparation

To minimize the effects of sampling variation and eliminate redundant data, we employed the software “EMNtools 1.0” to remove clustered samples within each 30 m × 30 m grid [45]. This procedure ensured that only a single occurrence point was retained per grid, resulting in a cumulative count of 151 valid occurrence records.

Collinearity among environmental variables can complicate the identification of key factors and lead to model overfitting [46]. To mitigate the effects of collinearity on the modeling process, we applied the Jackknife method in MaxEnt, using default parameters, to identify the most important variables influencing the model [47]. Subsequently, Pearson correlation analysis was conducted on 33 environmental variables associated with the species occurrence points using R version 4.1.2 [48]. Variables with a correlation coefficient greater than 0.8 were considered to be highly correlated [49]. Among the highly correlated variables, those contributing less to the prediction probability were excluded, and only the remaining variables were used for the final simulations [50].

### 2.4. Evaluation of Habitat Distribution in Current and Future Periods

To enhance the predictive accuracy of the MaxEnt model, the complexity can be reduced by adjusting default parameters such as the regularization multiplier (RM) and the feature combination (FC) settings [51,52]. In this study, we aimed to identify optimal combinations of RM and FC by initially setting the FC to LQHP and the RM to 1 as default parameters [53,54]. A systematic evaluation of the FC and RM parameter combinations was conducted using the “ENMeval” package in R [55]. The MaxEnt model utilizes five distinct feature types: linear (L), quadratic (Q), hinge (H), product (P), and threshold (T). Six FCs were adopted, namely L, H, LQ, LQH, LQHP, and LQHPT [55]. Eight RM values were tested, ranging from 0.5 to 4, with increments of 0.5 for each iteration [56]. The ENMeval tool evaluated 48 parameter combinations and selected the optimal configuration using the delta Akaike minimum information criterion corrected for small sample sizes (delta AICc) [53,54]. Models with delta AICc values < 2 were considered competitive, with lower delta AICc values indicating a better model fit. A higher delta AICc value suggests greater model accuracy [57], while the minimum delta AICc value of zero represents an optimal fit [58].

To assess the distribution of Reeves’s muntjac habitat under current and future scenarios. First, the filtered set of environmental variables associated with the occurrence points of Reeves’s muntjac in the current period were input into the MaxEnt model. The random test percentage was set at 25%, meaning that one-quarter of the occurrence data were used for model testing to verify its accuracy [49]. The remaining 75% of the data were used for model simulation [59]. We selected the “cut-off method to measure weight”, “Jackknife test to measure variable importance”, and “create response curve” options [60]. We subsequently optimized the RM and FC parameters, while keeping the remaining settings at their default values [54]. Habitat suitability maps for Reeves’s muntjac in the current period were generated after using 10 iterations of the modeling technique.

The accuracy of the model was evaluated using the area under the curve (AUC) of the receiver operating characteristic curve [61]. The AUC values range from 0 to 1, with higher values indicating greater predictive precision [17]. Generally, an AUC between 0.5 and 0.7 is considered poor, values between 0.8 and 0.9 are deemed acceptable, and values between 0.9 and 1.0 are considered excellent [52].

To project the future habitat suitability of Reeves’s muntjac, we assumed that all factors, except for climatic factors, would remain unchanged [47]. The current climatic factors in the MaxEnt model were replaced with climate projections for the 2050s and 2090s under the SSP126 and SSP585 scenarios, while all other simulation parameters were kept consistent with the current climate scenario [62]. The final habitat suitability maps for Reeves’s muntjac were generated after running the model for use 10 iterations.

### 2.5. Analysis of Climate Change-Induced Habitat Changes

The model’s simulation results are presented as grid layers representing the probability of species occurrence, with values ranging from 0 to 1 [61]. Higher values correspond to a greater likelihood of species occurrence [52]. To categorize habitat suitability, two thresholds were applied: the maximum test sensitivity plus specificity (MTSS) logistic threshold and the threshold value (TPT) logistic threshold, which balances training omission, predicted area, and threshold value. These thresholds were utilized to distinguish three suitability levels: low (L: 0.0 < TPT), medium (M: TPT < MTSS), and high (H: MTSS < 1.0) [63].

The functional zones of the NNR reflect both natural conditions and the extent of human activity [64,65]. The core zone represents minimal human activity and is subject to the strictest protection measures, while the experimental zone experiences the highest level of human activity and the least protection [66,67]. Compared to the core and experimental zones, the buffer zone exhibits moderate levels of both human activity and protection [68]. Consequently, the core and buffer zones are the primary distribution areas for Reeves’s muntjac habitat [22]. To verify the accuracy of the habitat suitability classification thresholds, we calculated the proportion of habitat areas at H, M, and L suitability levels within the three functional zones in the Hupingshan NNR. Upon validating the classification accuracy, we produced a habitat suitability map for Reeves’s muntjac. The future habitat suitability maps were then compared with current habitat suitability maps using ArcGIS to assess changes over time.

As a result of climate change, species may either remain in their current habitats, migrate to other suitable areas, or experience habitat loss [69]. To examine changes in the spatial distribution of Reeves’s muntjac habitats under projected climate change, we categorized habitat suitability into three levels: H, M, and L, represented by values of 2, 1, and 0, respectively. ArcGIS was used to analyze these distributions. The specific calculation procedure is as follows:

$$K=Habitat_{C}\times 10-Habitat_{F}$$

where $Habitat_{C}$ and $Habitat_{F}$ represent the habitat distribution results for Reeves’s muntjac in the current and future periods, respectively. The significance of the K-value is detailed in Table 2.

## 3. Results

### 3.1. Current Habitat Distribution

Using the default parameter settings for RM and FC in the MaxEnt model, the RM was set at 1, and the FC was LQPH, yielding a delta AICc value of 2085.9. After optimization, the RM and FC were adjusted to 2.5 and LQ, respectively, reducing the delta AICc value to 0. Therefore, the MaxEnt model parameters were set to an RM of 2.5 and an FC of LQ.

**Figure 2:**
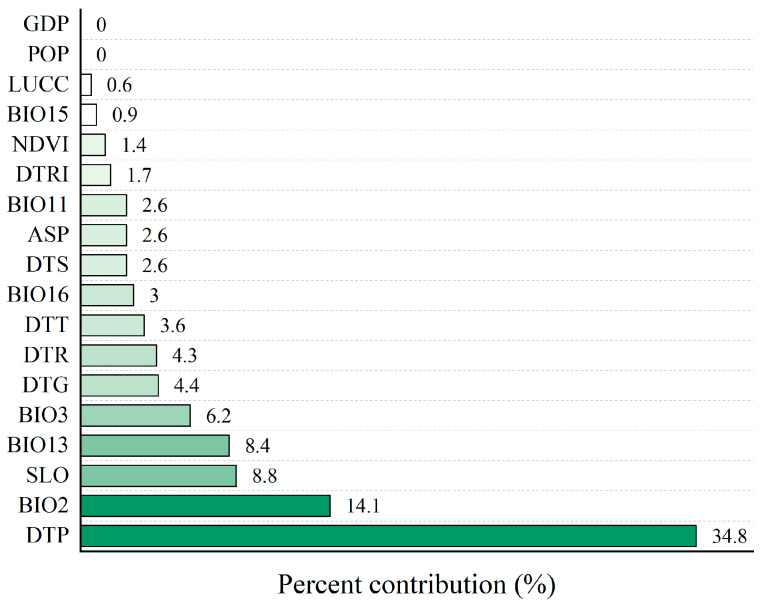
Influence of environmental variables on the distribution of Reeves’s muntjac habitat in Hupingshan NNR. GDP, gross domestic product; POP, population density; LUCC, land use/cover data; BIO15, precipitation seasonality; NDVI, normalized difference vegetation index; DTRI, distance to rivers; BIO11, mean temperature of the coldest quarter; ASP, aspect; DTS, distance to secondary roads; BIO16, precipitation of the wettest quarter; DTT, distance to tertiary roads; DTR, distance to residential roads; DTG, distance to government residences; BIO3, isothermality; BIO13, precipitation of the wettest month; SLO, slope; BIO2, mean diurnal air temperature range; DTP, distance to primary roads.

We excluded variables with minor contribution rates and those showing strong correlations. Ultimately, 18 key environmental variables influencing the distribution of Reeves’s muntjac were identified (Figure 2). Using 151 valid occurrence records and these 18 variables, the MaxEnt model was employed to assess the potential habitat distribution of Reeves’s muntjac in Hupingshan NNR. The model demonstrated strong predictive performance, with an AUC value of 0.84.

**Figure 3:**
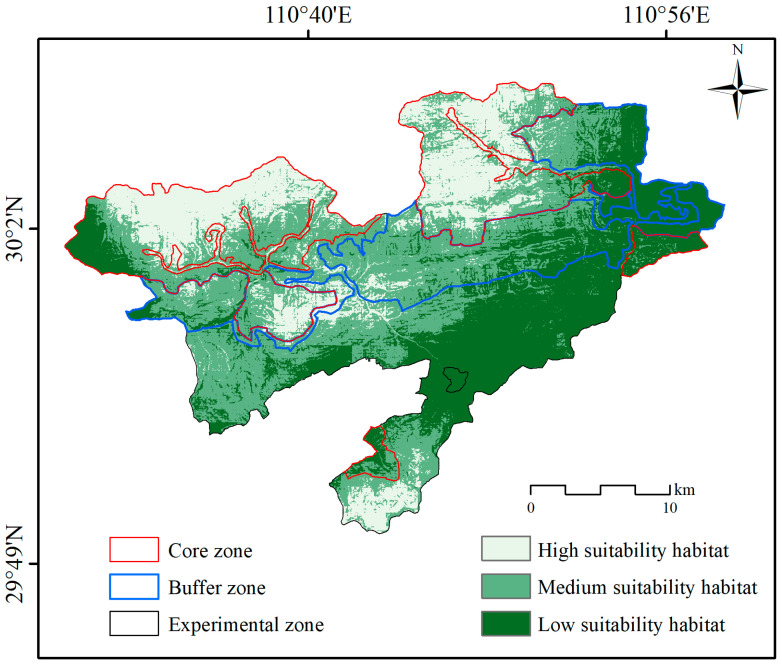
Current distribution of Reeves’s muntjac habitat in Hupingshan National Nature Reserve.

Figure 3 illustrates the current habitat distribution of Reeves’s muntjac habitat. Using the thresholds MTSS (0.3344) and TPT (0.0936), the habitat was categorized into three suitability levels. The areas of H, M, and L suitability habitats were 150.9 km^2^, 290.9 km^2^, and 225.9 km^2^, representing 22.6%, 43.6%, and 33.8% of the total study area, respectively.

The habitat distribution for Reeves’s muntjac across three functional zones during the current period is presented in Table 3. Under current conditions, the H suitability habitats within the core, buffer, and experimental zones cover an area of 114.5 km^2^, 11.5 km^2^, and 24.7 km^2^, representing 47.0%, 6.7%, and 9.9% of each zone’s total area, respectively. The M suitability habitats covered 95.0 km^2^, 95.3 km^2^, and 100.2 km^2^ in the core, buffer, and experimental zones, respectively, corresponding to 39.0%, 55.0%, and 40.0% of their respective areas. Finally, the L suitability habitats spanned 34.0 km^2^, 66.3 km^2^, and 125.4 km^2^ in these zones, accounting for 14.0%, 38.3%, and 50.1% of each zone’s total area, respectively.

The impact of environmental variables on the habitat distribution of Reeves’s muntjac is depicted in Figure 3. The primary contributing factors were the distance to primary roads (DTP) at 34.8%, the mean diurnal air temperature range (BIO2) at 14.1%, slope (SLO) at 8.8%, precipitation of the wettest month (BIO13) at 8.4%, isothermality (BIO3) at 14.1%, and the distance to government residences (DTG) at 4.4%. These six environmental factors collectively contributed 76.3%, indicating that they are the key drivers of habitat selection for Reeves’s muntjac, while the remaining 12 variables contributed 23.7%. Furthermore, climatic factors—BIO15, BIO11, BIO16, BIO3, BIO13, and BIO2—collectively contributed 35.2%, and human disturbance factors, including GDP, POP, LUCC, DTRI, DTS, DTT, DTR, DTG, and DTP, contributed 49.4%.

**Figure 4:**
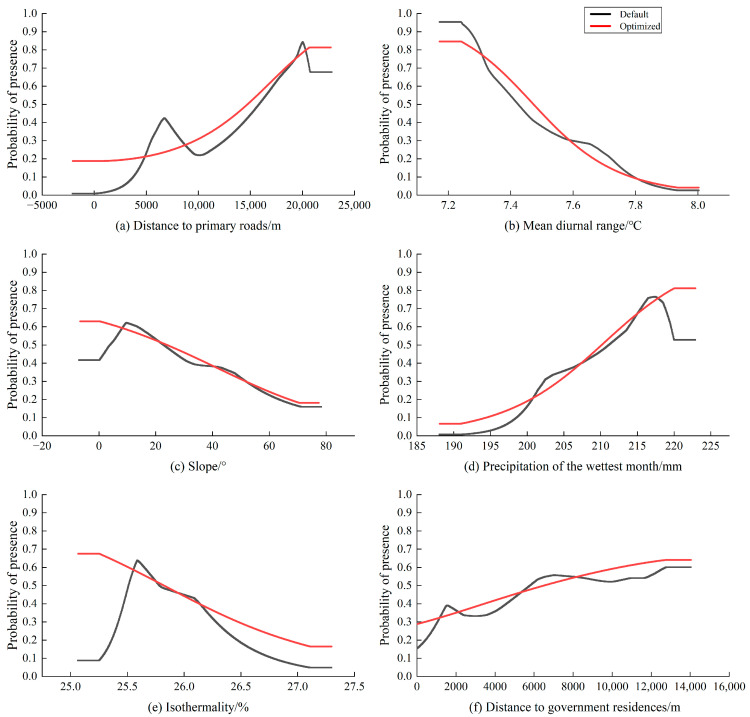
Response curves illustrating the impact of the six most significant environmental factors on habitat suitability of Reeve’s muntjac.

Logistic regression analysis within the MaxEnt model was employed to explore the relationships between the probability of habitat distribution and six key environmental parameters. Figure 4 illustrates the response curves of these variables, highlighting their impact on the survival of Reeves’s muntjac. Habitat areas were classified as those with a distribution probability greater than 0.5. When considering the influence of individual environmental factors, “suitable habitats” refer to regions that meet specific criteria: a minimum distance of 14.72 km from primary roads, a mean diurnal range below 7.5 °C, a slope of no more than 24°, precipitation during the wettest month of at least 210.2 mm, an isothermality value under 25.8%, or a distance of at least 6.5 km from government residences.

### 3.2. Future Habitat Distribution

**Figure 5:**
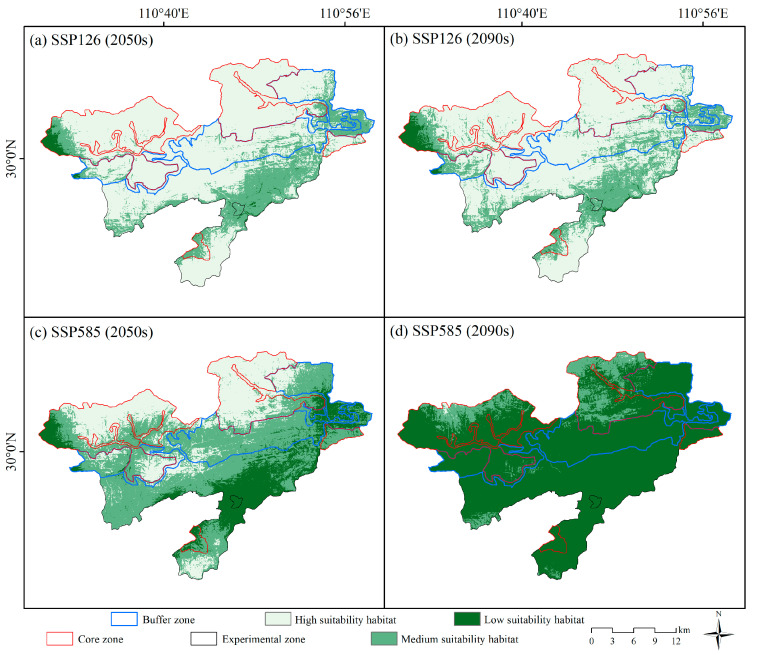
Habitat suitability maps for Reeves’s muntjac under two shared socioeconomic pathways (SSP126 and SSP585) for the mid-century (2050s) and the late-century (2090s).

Figure 5 presents the habitat suitability maps of Reeves’s muntjac for the future periods. The areas of H, M, and L suitability habitats under different climate scenarios are listed in Table 4. Under the SSP126 scenarios, the H, M, and L suitability habitats in the 2050s covered 500.5 km^2^, 157.5 km^2^, and 9.7 km^2^, respectively. By the 2090s, these areas slightly decreased to 482.2 km^2^ for H suitability habitats and increased to 169.1 km^2^ and 16.4 km^2^ for M and L suitability habitats, respectively. Conversely, under the SSP585 scenarios, the total area of H, M, and L suitability habitats in the 2050s spanned 203.9 km^2^, 336.8 km^2^, and 127.0 km^2^, respectively. By the 2090s, a dramatic shift was observed, with the area of H suitability habitats plummeting to 1.5 km^2^ and that of M and L suitability habitats rising to 72.1 km^2^ and 594.1 km^2^, respectively.

### 3.3. Climate Change-Induced Habitat Changes Under Different Future Periods

**Figure 6:**
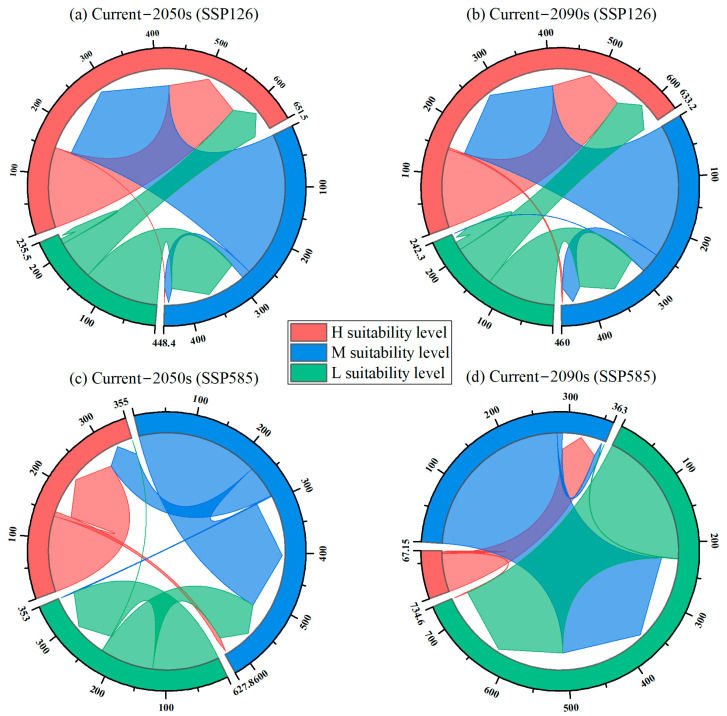
Comparison of changes in habitat area suitability levels (H: high, M: medium, and L: low) for Reeve’s muntjac across current and future periods, based on two shared socioeconomic pathways (SSP126 and SSP585) for the mid-century (2050s) and the late-century (2090s).

Figure 6 illustrates the changes in habitat suitability for Reeves’s muntjac under future climate scenarios compared to the current period. The extent of changes in habitat suitability levels for future periods compared to the current levels is detailed in Table 5.

Under the SSP126 scenario in the 2050s, the unchanged areas of H, M, and L suitability habitats were 150.9 km^2^, 16.0 km^2^, and 9.7 km^2^, respectively, with corresponding K-values of 18, 9, and 0. However, 274.9 km^2^ of habitat transitioned from M to H suitability, 141.5 km^2^ from L to M suitability, and 74.7 km^2^ from L to H suitability levels. By the 2090s, under SSP126, the unchanged areas of H, M, and L suitability habitats were 147.4 km^2^, 37.2 km^2^, and 15.7 km^2^, respectively, with corresponding K-values of 18, 9, and 0. However, 3.5 km^2^ of suitable habitat shifted from H to M suitability, 0.7 km^2^ from M to L suitability, 253.0 km^2^ from M to H suitability, 128.4 km^2^ from L to M suitability, and 81.8 km^2^ from L to H suitability levels, with corresponding K-values of 19, 10, 8, −1, and −2, respectively.

In the 2050s, under the SSP585 scenario, the unchanged areas of H, M, and L suitability habitats were 142.6 km^2^, 227.8 km^2^, and 125.1 km^2^, respectively, with corresponding K-values of 18, 9, and 0. However, 8.3 km^2^ of habitat transitioned from H to M suitability, 1.9 km^2^ from M to L suitability, 61.2 km^2^ from M to H suitability, 100.7 km^2^ from L to M suitability, and 0.1 km^2^ from L to H suitability, with corresponding K-values of 19, 10, 8, −1, and −2. In the 2090s, under the SSP585 scenario, the unchanged areas of H, M, and L suitability habitats were 1.5 km^2^, 8.5 km^2^, and 224.8 km^2^, respectively, with corresponding K-values of 18, 9, and 0. However, 86.9 km^2^ of habitat transitioned from H to L suitability, 62.5 km^2^ from H to M suitability, 282.4 km^2^ from M to L suitability, and 1.1 km^2^ from L to M suitability, with corresponding K-values of 20, 19, 10, and −1, respectively.

## 4. Discussion

### 4.1. Selection of Environmental Factors

The habitat preferences of a species can vary significantly across different environments [70], and given the limited research on Reeve’s muntjac habitat selection in the Hupingshan NNR, we assumed that the species exhibits similar habitat preferences to those observed in other regions. To model the habitat distribution of Reeve’s muntjac, we selected 33 environmental variables based on previous studies that investigated habitat preferences in other locations (Table 1). Our results indicated that anthropogenic activities, climate, and terrain are the primary drivers influencing the habitat distribution of Reeve’s muntjac in the Hupingshan NNR (Figure 3). These factors collectively accounted for 96.3% of the variability in habitat suitability. This suggests that an integrated approach considering these three key environmental factors is essential for accurately assessing suitable habitats for the species within this region. These findings are consistent with similar habitat preferences for Reeve’s muntjac in the Houhe NNR [36], as well as in the Qingliangfeng NNR [35].

However, our results revealed that the overall contributions of vegetation and hydrological factors to habitat suitability for Reeve’s muntjac were relatively low, at only 3.7% (Figure 3). This contrasts with Reeves’s muntjac habitat selection in eastern England [40]. This disparity may be attributed to several factors. First, the high vegetation coverage and favorable growth conditions in the Hupingshan NNR likely meet the species’ needs for food and shelter throughout the reserve. Second, restricted human activities in the core and buffer zones further enhance habitat stability, allowing Reeve’s muntjac to occupy a wider range of vegetative areas. Third, the diverse topography and micro-climates within the reserve likely create abundant ecological niches, further buffering against potential limitations imposed by vegetation availability. Furthermore, the minimal influence of hydrological factors, that is, water sources, on habitat suitability differs from the findings of the Qinghai-Tibet Plateau, where a greater dependence on water availability was observed [13]. This variation could be explained by the abundant water resources and precipitation in the Hupingshan NNR, which likely fulfill the water requirements of Reeve’s muntjac, rendering water availability a non-limiting factor for their activity.

### 4.2. Analysis of Major Environmental Factors Affecting Habitat Selection

This study identified six key environmental factors influencing the distribution of the Reeve’s muntjac habitat, ranking by their contribution (from highest to lowest): distance to primary roads (DTP), mean diurnal air temperature range (BIO2), slope (SLO), precipitation of the wettest month (BIO13), isothermality (BIO3), and distance to government residences (DTG).

Both primary roads and government residences, along with their surrounding areas, represent zones of high anthropogenic activity within the Hupingshan NNR [71]. It was observed that Reeve’s muntjac tends to maintain a significant distance from human presence, specifically ≥14.72 km from primary roads and ≥6.5 km from government residences (Figure 4). This behavior is likely due to their timid nature and aversion to large structures and disturbances. The greater distance from primary roads compared to government residences suggests that human activity or hunting (poaching) may be more prevalent along primary roads, and the construction of additional primary roads or residences within the NNR should be restricted [72].

The slope of the suitable habitat for Reeve’s muntjac is generally ≤24°, likely because gentler slopes reduce energy expenditure during migration [12]. However, the research conducted in the Qingliangfeng NNR suggests that slope has minimal influence on Reeve’s muntjac survival [35], which contrasts with the findings of this study. The discrepancy may be attributed to the lower slope variability in the Qingliangfeng region, where such topographic factors are less influential on habitat selection.

Reeve’s muntjac shows a preference for habitats where the average daily temperature is ≤7.5 °C, as smaller diurnal temperature variations help minimize the energy required to maintain a constant body temperature [73]. In addition, the isothermality of suitable habitats is ≤25.8%, suggesting that Reeve’s muntjac thrives in areas with greater annual temperature fluctuations. This adaptability to seasonal changes is a key survival strategy, as they adjust their behavior and metabolic processes accordingly. During resource-rich summers, they breed and build up energy reserves, while in cold winters, they reduce activity or modify foraging behaviors to conserve energy [12,43].

Reeve’s muntjac prefers habitats with ≥210.2 mm of precipitation during the wettest month. This preference is likely linked to their herbivorous diet, as the wettest season in southern China, typically summer, coincides with periods when food resources are relatively scarce [74]. Abundant rainfall promotes the growth of mosses and fungi, which serve as important food sources for the muntjacs [16]. Additionally, the high summer temperatures are mitigated by rainfall, which helps lower the temperature [75]. This cooling effect benefits the muntjacs by reducing the evaporation of body moisture, thus aiding in maintaining their water balance [76].

This study further suggests that altitude is not a limiting factor for habitat selection of Reeve’s muntjac in Hupingshan NNR, likely due to restrictions on human activities within the reserve, which reduce anthropogenic pressures across altitudinal gradients. This finding is consistent with the southern Qinling region of Shaanxi Province [77]. However, in Qingliangfeng NNR, Reeve’s muntjac favored altitudes above 800 m, a difference likely attributable to the distinct socio-economic characteristics and environmental pressures of the surrounding areas in the region [11]. In mid- to low-altitude, and valley regions, anthropogenic activity, livestock grazing, and the presence of domestic dogs often create disturbances that drive Reeve’s muntjac to seek refuge in higher-altitude areas to avoid these threats [78].

### 4.3. Impact of Climate Change

The results of this study under the SSP126 climate scenario suggest an expansion in the total area of suitable habitats for Reeve’s muntjac in both the 2050s and 2090s, compared to the current climate. In this scenario, the projected temperature increase remains moderate and within the physiological tolerance range of Reeve’s muntjac [79]. A moderate rise in temperature can enhance plant photosynthesis, leading to improved vegetation growth [80], which in turn benefits habitat suitability by expanding the available forage and cover. However, in the SSP585 scenario, the significant rise in temperature poses a potential threat to the distribution of Reeve’s muntjac habitats. While the temperature increases projected for the 2050s may still favor the species’ survival, allowing for habitat expansion, the scenario changes drastically by the 2090s. At this point, the continuous and severe temperature rise leads to drastic environmental alterations that surpass the physiological tolerance of Reeve’s muntjac [81].

In contrast, in southern China indicates that the habitat area for Reeve’s muntjac responds positively to climate change, with potential increases in habitat areas under various climate change scenarios [4]. For example, in the six major mountain ranges of China—Qinling, Minshan, Qionglai, Daxiangling, Xiaoxiangling, and Liangshan—the response of Reeve’s muntjac habitats to climate change has decreased across different climate scenarios [82]. Furthermore, the Mediterranean region may become even less favorable for the Reeve’s muntjac as it warms and becomes drier [16]. Finally, many parts of northern Europe, particularly along the northwest Atlantic coast (northern France to Scandinavia), might become more favorable for the species, especially where increased average temperatures and precipitation continue to support the growth of woodlands and forests [83].

While numerous studies concur that climatic factors play a crucial role in the habitat selection of Reeve’s muntjac [4,12,13,14,16,36,44], there remains considerable debate regarding whether climate change will have a positive or negative impact on their suitable habitat areas. This divergence of opinion may be attributed to two main factors. First, different studies employ varying models for simulating and predicting future climate conditions. For instance, while this study used the BCC-CSM2-MR model of the CMIP6 framework, Sun et al. [4] applied the MIROC5 model from CMIP5, and Li [82] utilized the MRI-ESM2-0 model from CMIP6. These models can yield different projections, particularly when simulating regional climate changes, leading to varying conclusions about the impacts on Reeve’s muntjac habitats. Second, discrepancies in habitat suitability assessment methods contribute to the ongoing debate. Sun et al. [4] and Li [82] both used the MaxEnt model to evaluate habitat suitability [4,82], while Ward et al. [83] employed correlation analysis.

In summary, the impact of climate change on the habitat of Reeve’s muntjac remains a complex and contentious issue, requiring further, more detailed exploration. Future studies should consider incorporating multiple models and methods to achieve more comprehensive and reliable conclusions.

### 4.4. Protection of the South China Tiger

Given the extinction of the South China tiger in the wild, directly studying its suitable habitat distribution in the Hupingshan NNR is not feasible. However, research indicates a significant positive correlation between the number and distribution of large predators, such as the South China tiger, and that of their prey species [84]. In this study, we assessed the distribution of suitable habitats for Reeve’s muntjac, a key prey species of the South China tiger. This can provide valuable insight into the proposed reintroduction of the South China tiger into Hupingshan NNR. However, it is essential to recognize that this study focused solely on Reeve’s muntjac habitats. Other prey species, such as Wild boar and Tufted deer, also inhabit the Hupingshan NNR and are crucial for a sustainable tiger population [22]. To develop a comprehensive and effective conservation strategy, future studies should assess the habitat distributions of all major prey species. Only through this holistic approach can we accurately determine the suitable habitats for the South China tiger and devise more informed and scientifically robust reintroduction plans.

### 4.5. Additional Considerations and Implications

This study assumes that non-climatic environmental factors within the Hupingshan NNR will remain constant over the next century. In reality, these factors are likely to be affected by ongoing environmental changes and human activities [85]. Therefore, our findings may underestimate the potential impact of future human development on Reeve’s muntjac habitats, and by extension, the habitat suitability for South China tigers. Thus, targeted measures to protect and bolster Reeve’s muntjac numbers are essential. For instance, strengthened anti-poaching efforts, habitat restoration initiatives, community-based conservation programs, and measures to restrict the construction of additional primary roads or residences within the NNR can help mitigate the negative effects of human activities on wildlife populations. Furthermore, ongoing monitoring and adaptive management strategies will be necessary to account for these uncertainties in habitat conservation efforts.

Additionally, we used 1970–2000a climate data as the “current” baseline, a common practice in many ecological models for consistency and comparability. However, this does not fully capture the climate data of 2000–2024a. Future research could utilize updated climate datasets to refine the habitat distribution of Reeve’s muntjac in the current period.

Finally, only one year of field data were used to evaluate the habitat distribution of Reeve’s muntjac. Prolonged monitoring periods across multiple years would yield a more comprehensive assessment of the habitat distribution of Reeve’s muntjac.

## 5. Conclusions

Our findings highlight that climatic and human disturbance factors are the two primary determinants influencing the habitat distribution of Reeve’s muntjac in the current period. Although non-climatic environmental factors were assumed to remain constant over the next century, future climate change is likely to reduce habitat suitability and increase fragmentation, thereby elevating the extinction risk for Reeves’s muntjac. Effective measures such as constructing migration corridors, minimizing human activity, restricting further infrastructure development, or even removing existing infrastructures, such as government residences, should be considered to mitigate the adverse effects of climate change and ensure the success of the South China tiger reintroduction program, which relies on adequate prey availability. However, due to the assumption of constant human activities and the exclusion of the climate change impacts on other prey species of tigers, our findings may underestimate the potential impact of future human development on Reeve’s muntjac habitats, and consequently, on the habitat suitability for South China tigers. Continuous monitoring and adaptive management strategies will be essential to address these uncertainties and support effective habitat conservation.

## Figures and Tables

**Table 1:** Environmental variables and their descriptions.

Category	Variable	Description	References
Vegetation factor	NDVI	Normalized difference vegetation index	[11,12,13,14,16,36,40,41,42,43,44]
	LUCC	Land use/cover data	
Topographic factor	ELE	Elevation	[4,11,12,13,14,16,36,41,42,43,44]
	ASP	Aspect	
	SLO	Slope	
Human activity factor	DTP	Distance to primary roads	[11,13,14,35,36,41,42,43,44]
	DTS	Distance to secondary roads	
	DTT	Distance to tertial roads	
	DTR	Distance to residential roads	
	LUCC	Land use/cover data	
	GDP	Gross domestic product	
	POP	Population density	
	DTG	Distance to government residences	
Hydrological factor	DTRI	Distance to rivers	[11,12,13,35,36]
	DTWB	Distance to water bodies	
Climatic factor	BIO1	Annual mean temperature	[4,12,13,14,16,36,44]
	BIO2	Mean diurnal air temperature range	
	BIO3	Isothermality	
	BIO4	Temperature seasonality	
	BIO5	Maximum temperature of the warmest month	
	BIO6	Minimum temperature of the coldest month	
	BIO7	Temperature annual range	
	BIO8	Mean temperature of the wettest quarter	
	BIO9	Mean temperature of the driest quarter	
	BIO10	Mean temperature of the warmest quarter	
	BIO11	Mean temperature of the coldest quarter	
	BIO12	Annual precipitation	
	BIO13	Precipitation of the wettest month	
	BIO14	Precipitation of the driest month	
	BIO15	Precipitation seasonality	
	BIO16	Precipitation of the wettest quarter	
	BIO17	Precipitation of the driest quarter	
	BIO18	Precipitation of the warmest quarter	
	BIO19	Precipitation of the coldest quarter	

**Table 2:** Interpretation of K-value and changes in future habitat suitability levels for Reeve’s muntjac compared to current levels.

Number	K-Value	Significance	Changes in Future Habitat Suitability Levels Compared to Current Levels
1	20	Area is highly suitable in the current period but less suitable in the future	Descend (↓)
2	19	Area is highly suitable in the current period but moderately suitable level in the future	Descend (↓)
3	18	Area remains highly suitable in both current and future periods	Unchanged (―)
4	10	Area is moderately suitable in the current period but less suitable in the future	Descend (↓)
5	9	Area remains moderately suitable in both current and future periods	Unchanged (―)
6	8	Area is moderately suitable in the current period but highly suitable in the future	Ascend (↑)
7	0	Area remains less suitable in both current and future periods	Unchanged (―)
8	−1	Area is less suitable in the current period but moderately suitable in the future	Ascend (↑)
9	−2	Area is less suitable in the current period but highly suitable in the future	Ascend (↑)

**Table 3:** Area and proportion of suitable habitats for Reeves’s muntjac under the current period in the core, buffer, and experimental zones of Hupingshan National Nature Reserve.

	Core Zone		Buffer Zone		Experimental Zone		Total Area/km^2^	Proportion/%
	Area/km^2^	Proportion/%	Area/km^2^	Proportion/%	Area/km^2^	Proportion/%		
High suitability area	114.5	47.0	11.5	6.7	24.7	9.9	150.9	22.6
Medium suitability area	95.0	39.0	95.3	55.0	100.2	40.0	290.9	43.6
Low suitability area	34.0	14.0	66.3	38.3	125.4	50.1	225.9	33.8

**Table 4:** Area of high, medium, and low suitability habitats for Reeves’s muntjac under two shared socioeconomic pathways (SSP126 and SSP585) for the mid-century (2050s) and the late-century (2090s).

	SSP126		SSP585	
	2050s	2090s	2050s	2090s
	Area/km^2^	Area/km^2^	Area/km^2^	Area/km^2^
High suitability habitat	500.5	482.2	203.8	1.5
Medium suitability habitat	157.5	169.1	336.9	72.1
Low suitability habitat	9.7	16.4	127.0	594.1

**Table 5:** The area change in habitat suitability levels for Reeve’s muntjac under two shared socioeconomic pathways (SSP126 and SSP585) for the mid-century (2050s) and the late-century (2090s) compared to the current habitat suitability level.

K-Value	SSP126		SSP585	
	2050s	2090s	2050s	2090s
	Area/km^2^	Area/km^2^	Area/km^2^ (2050s)	Area/km^2^ (2090s)
20	0	0	0	86.9
19	0	3.5	8.3	62.5
18	150.9	147.4	142.6	1.5
10	0	0.7	1.9	282.4
9	16.0	37.2	227.8	8.5
8	274.9	253.0	61.2	0
0	9.7	15.7	125.1	224.8
−1	141.5	128.4	100.7	1.1
−2	74.7	81.8	0.1	0.0

## Data Availability

The data presented in this study are available upon request from the corresponding author. The data are not publicly available due to the constraint in the consent.
